# Soft skills in personnel training: Report of publications in scopus, topics explored and future research agenda

**DOI:** 10.1016/j.heliyon.2023.e15468

**Published:** 2023-04-14

**Authors:** Lorena C. Espina-Romero, Sandra Lucia Aguirre Franco, Helga Ofelia Dworaczek Conde, Jesús M. Guerrero-Alcedo, Doile Enrique Ríos Parra, Juan Carlos Rave Ramírez

**Affiliations:** a Escuela de Postgrado, Universidad San Ignacio de Loyola, Lima, Peru; b Unidad Central del Valle del Cauca, Colombia; c Universidad Santo Tomás, Colombia; d Universidad Científica del Sur, Lima, Peru; e Universidad Popular del Cesar (UPC), Colombia; f Universidad del Quindío, Colombia

**Keywords:** Employment, Human resource management, Soft skills, Staff training, Technical skills

## Abstract

Recent research has documented the interest of organizations in training their staff in soft skills, but few studies have been found. Therefore, the objective of this research was to analyze 753 publications in the Scopus database related to soft skills in staff training during the period 1999–2021. These documents were analyzed to identify the main information, the most explored areas, and a future research agenda; all under a bibliometric and bibliographic approach with the help of RStudio and VOSviewer software. The results showed that the keywords with the most co-occurrence were personnel training (n = 110) and soft skills (n = 79). The year with the most documents was 2021 (n = 121). The country with the most publications was the United Kingdom (n = 199). Medicine is the subject area with the most documents (n = 278) and the Article is the type of document with the most studies (n = 566). Eleven areas of further exploration were identified: “Soft skills in software engineering at the higher education level”, “Soft skills and communication”, “Soft skills and engineering education”, “Soft skills in virtual environments”, “Soft skills in machine learning”, “Serious games in teaching soft skills”, “Soft skills for problem-based learning”, “Soft skills for project management”, “Soft skills and technical skills”, “Project-based learning for the assessment of soft skills” and “Soft leadership skills”. Five potential areas for future research were derived: soft skills in collaborative work (CSCL), soft skills in computer-aided collaborative work (CSCW), facial expressions as a mirror of soft skills, soft skills for employability and Professional Development Plan (PDP) to assess soft skills. In conclusion, this Review type document on soft skills in personnel training helped to identify the most studied topics during the evaluated period, as well as to identify the little explored topics for future research.

## 1 Introduction

When social skills are coupled with those of communication, those of way of being, those of approach and others, they result in the so-called soft skills and are those that mold people in their ability to communicate and relate appropriately with others; becoming a factor highly valued by organizations when it comes to training their staff. There are numerous soft skills that organizations value when training staff, among them are tolerance to pressure, organization and planning, honesty and professional ethics, ability to adapt to changes, capacity for empathy, positive attitude, proactivity, communication skills, conflict resolution and one of the most fundamental that is teamwork [1].

The quality of professionals in the labour market, their rapid adaptation to the demands of employers or the possible change of specialization in the short term, are increasingly necessary and important attributes [2,3]. Now, soft skills have become a very important component in organizations, highlighting technical skills which are not considered difficult to develop, but getting qualified professionals with less than a year of graduation and who possess the soft skills necessary for an organization, is somewhat difficult [[4], [5], [6]]. Therefore, employers are looking for more applicants with great special skills, because it is easier to train a staff in soft skills than in hard skills [7,8].

For the field of applied neuroscience in education, learning soft skills such as empathy, cognitive control, and flexibility are essential. That is, education cannot be considered only to study hard subjects [9]. However, soft skills can be worked in a similar way to how hard skills are worked in various fields, and it is thanks to gamification that it is a widely used playful tool, which guarantees to work one or more soft skills [10].

The main research topics addressed by soft skills are oriented towards staff training [11], engineering education [12], human resource management [13], project management [14], communication [15], decision making [2], problem solving [16], professional development [17] and curricula [1], among others.

After reviewing the literature, it was possible to find the study by Fernandez-Arias et al. [18], which was based on the design of a survey to analyze the development of soft skills in a group of 284 Latin American engineers and describe the gap between men and women. The findings showed high ratings of their soft skills and gender differences in work behavior and social skills in favor of women.

Another recent study is the one carried out by Pota et al. [19], who propose a decision model to evaluate the effectiveness of the level of shared satisfaction in task assignments under the direction of human resources. The characteristics of the model are a) combination of soft skills of employees with hard skills, b) self-assessment of interpersonal skills of workers, c) joint self-assessment between managers and workers on which tasks they prefer to perform, and d) measurement by means of metrics of the coincidences between the characteristics, desires of the employees and their jobs.

On the other hand, Dahl [20] prepared a manuscript whose objective was to provide a bibliographical synopsis of the themes in the use of immersive virtual learning and the formation of interpersonal skills of workers. The findings showed that there is a lack of bibliographic studies and empirical research in this regard.

Likewise, the study by Sultanova et al. [21] considered the difficulty in the development of soft skills of Mathematics and Physics scholars of institutions at the university level during a postgraduate certification of the Ukrainian educational system. The experience of other countries regarding the nature and relevance of soft skills such as sociability, creativity and empathy was analyzed. Options for developing interpersonal skills were set. The four-stage process of their transition from involuntary incompetence to involuntary competence was justified at the graduate level.

Another “Review” type study related to soft skills in staff training, which is called “Assessing the demand for soft skills in software development” was prepared by Ahmed et al. [22]; This study looked at which soft skills cited in 500 information technology job ads were in significant demand for software development and which were not. Although this review study provides essential information on soft skills linked to staff training, it does not provide an overview of the development of this topic. It only indicates the historical evolution and trend topics during 1999–2013. Therefore, it is important to carry out a more up-to-date analysis on the subject from 1999 to the date of the preparation of this study (December – 2021) and that is achieved with the preparation of a bibliometric review to know about this topic, which areas have been studied and which fields remain unexplored.

In line with the above, this study raises three research questions.

RQ1 What is the main information available on Scopus on soft skills in staff training?

RQ2 What are the most explored topics?

RQ3 What could be the potential topics of future research?

To answer the three questions, a bibliometric review was carried out with the main objective of analyzing the publications indexed in the Scopus database on soft skills in the training of personnel during the period 1999–2021, all under a bibliographic approach.

## 2 Methodology

**Fig. 1:**
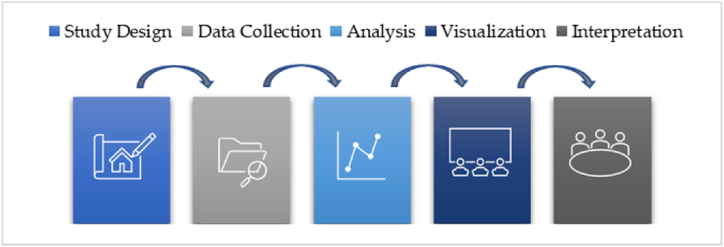
Methodological design.

This study carries out a bibliometric review and this is used in different scientific fields gaining importance among academics [23]. For Zupic and Čater [24], there are five steps for its realization and consist of: 1) study design, 2) collection, 3) analysis, 4) visualization and 5) interpretation (Fig. 1).

**Study Design:** After the literature review, three research questions (RQ1, RQ2 and RQ3) were formulated at the end of the Introduction section. To answer the.

• RQ1: The bibliometric method “keyword co-occurrence analysis” was chosen to indicate the keywords with the highest co-occurrence. On the other hand, Microsoft Excel column charts were used to indicate the annual production of documents, the production of publications by country/territory, number of documents by thematic area, and number of manuscripts by type of document.
• RQ2: The Thematic Map was chosen, considered as a bibliometric method of conceptual structure. This indicates the directions of the topics associated with the soft skills.
• RQ3: For this question, the bibliometric method “keyword co-occurrence analysis” was used again, but this time the keywords with less co-occurrence were used to infer the associated topics that would serve as a guide for future studies.

**Collection of bibliometric data**: The reason for choosing Scopus as the only database is because it has extensive coverage over time and houses more than 40,000 journals that cover all subject areas, in addition to this, this platform is updated regularly, allowing access to recent knowledge. We proceeded to enter the terms “soft skills” or “personal skills” or “interpersonal skills” or “non-technical skills” or “essential skills” or “transferable skills” and “personnel training” or “people” or “staff” or “manpower” or “human resources” in the search field labeled “article title/abstract/keywords”, and this returned 3704 documents. The search was then limited to “All Open Access” yielding 951 records. Subsequently, it was limited to the period 1999–2021, yielding 765 records. Note, Short Survey and Editorial type documents were excluded, obtaining 757 records. Articles in Press were excluded, obtaining 753 publications. No country or subject area was excluded.

As a result, the following search string was generated: “TITLE-ABS-KEY (“Soft skills” OR “personal skills” OR “interpersonal skills” OR “non-technical skills” OR “essential skills” OR “transferable skills” AND “personnel training” OR “people” OR “staff” OR “manpower” OR “human resources”) AND (LIMIT-TO (OA, “all")) AND (LIMIT-TO (PUBYEAR, 2021) OR LIMIT-TO (PUBYEAR, 2020) OR LIMIT-TO (PUBYEAR, 2019) OR LIMIT-TO (PUBYEAR, 2018) OR LIMIT-TO (PUBYEAR, 2017) OR LIMIT-TO (PUBYEAR, 2016) OR LIMIT-TO (PUBYEAR, 2015) OR LIMIT-TO (PUBYEAR, 2014) OR LIMIT-TO (PUBYEAR, 2013) OR LIMIT-TO (PUBYEAR, 2012) OR LIMIT-TO (PUBYEAR, 2011) OR LIMIT-TO (PUBYEAR, 2010) OR LIMIT-TO (PUBYEAR, 2009) OR LIMIT-TO (PUBYEAR, 2008) OR LIMIT-TO (PUBYEAR, 2007) OR LIMIT-TO (PUBYEAR, 2006) OR LIMIT-TO (PUBYEAR, 2005) OR LIMIT-TO (PUBYEAR, 2004) OR LIMIT-TO (PUBYEAR, 2003) OR LIMIT-TO (PUBYEAR, 2002) OR LIMIT-TO (PUBYEAR, 2001) OR LIMIT-TO (PUBYEAR, 2000) OR LIMIT-TO (PUBYEAR, 1999)) AND (LIMIT-TO (DOCTYPE, “ar”) OR LIMIT-TO (DOCTYPE, “cp”) OR LIMIT-TO (DOCTYPE, “re”) OR LIMIT-TO (DOCTYPE, “ch")) AND (LIMIT-TO (PUBSTAGE, “final"))”.

**Data analysis:** In this step, the information is loaded into the statistical packages for its conversion to guarantee its quality without forgetting its validity. The information was collected from Scopus in CSV format to be processed by the software VOSviewer version 1.6.18, RStudio version R 4.1 0.1 and the Microsoft Excel 365 web application.

**Data visualization:** This study was supported by column charts generated by the Microsoft Excel 365 web application to indicate the production of manuscripts by year, country, subject area, and type of document. On the other hand, it was based on the Thematic Map of the RStudio software to visualize the study topics associated with soft skills. At the same time, it was based on the bibliometric method “keyword co-occurrence analysis” of the VOSviewer software to visualize the keywords with the highest co-occurrence and associated topics that can shape a future research agenda.

**Interpretation:** Here the results are described and represented according to the objectives, i.e., the main information, the topics associated with soft skills and the associated topics destined for a future research agenda are discussed.

## 3 Results and discussions

### 3.1 Main information

#### 3.1.1 Co-occurrence of keywords

**Fig. 2:**
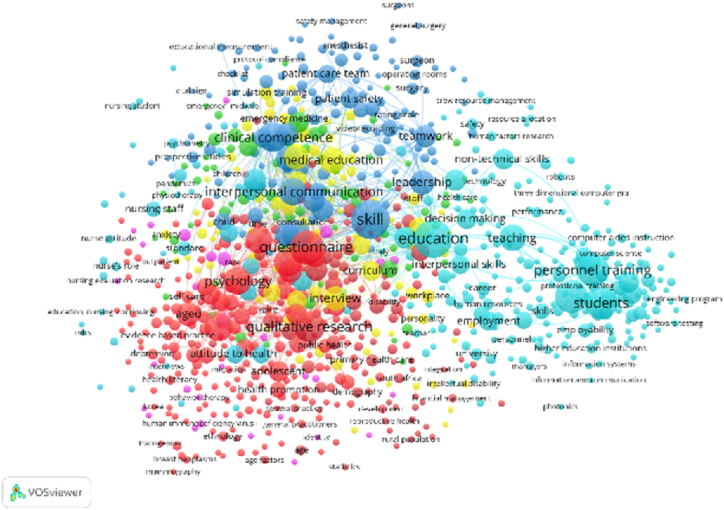
Co-occurrence of keywords (VOSviewer – scopus).

In the 753 publications selected for the present review study, a total of 4971 Keywords were totaled, of which 32 items were selected as the words with the most co-occurrence, all of them grouped into 5 clusters of different colors as shown in Fig. 2.

In the red cluster made up of 10 items, the keyword of more co-occurrence is personnel training (n = 110), closely related to the words soft skills (n = 79), technical skills (n = 12), communication (n = 45), online learning (n = 9), information systems (n = 30), computer science (n = 6), non-technical skills (n = 33), training (n = 35) and virtual reality (n = 10), which allows us to infer that organizations demand training programs for the critical development of the capabilities of their staff, especially in the management of information [14,25,26].

For the green cluster composed of 9 keywords, the one with the highest co-occurrence is employment (n = 36) with close link to the words project management (n = 12), surveys (n = 13) communication skills (n = 24), human resource management (n = 28), soft skills development (n = 9), decision making (n = 27), managers (n = 9) and societies and institutions (n = 6), which allows us to deduce that soft skills have managed to obtain value and importance in the world of work, because they work to solve problems and thus face their working time in an appropriate way [27,28].

In the blue cluster, 6 items are grouped, where the word for the most co-occurrence is students (n = 107) with a close relationship to education in engineering (n = 52), education (n = 80), curriculum (n = 33), teaching (n = 29) and computer education (n = 13), deducing that although the teaching of certain social skills is not contemplated in the educational curricula, these are very important in the preparation of students for inclusion in the jobs of the future [13,29].

For the yellow cluster with 6 items, the keyword it leads is software engineering (n = 28) linked to words such as professional aspects (n = 15), problem solving (n = 14), software design (n = 13), interpersonal skills (n = 78) and engineers (n = 8), allowing us to infer that there is a challenge in the field of software engineering in creating tools for applications that allow the evaluation of soft skills in the training of personnel [30]. Finally, the purple color cluster with the word identified as learning systems (n = 11).

#### 3.1.2 Publications by year

**Fig. 3:**
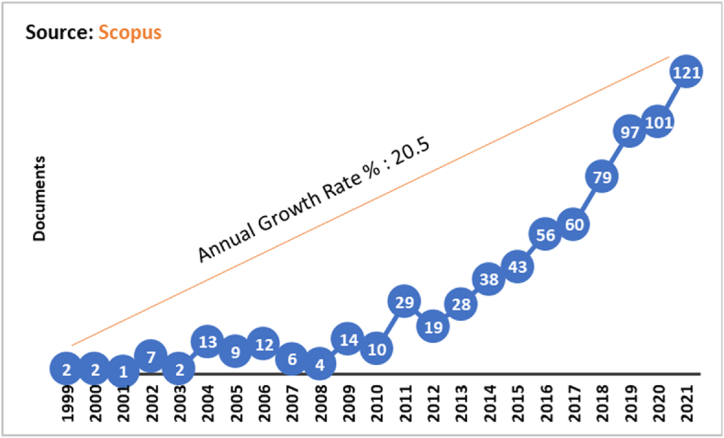
Documents per year.

The annual growth rate during the period 1999–2021 is 20.5%. Fig. 3 shows the years 1999 and 2000 with only 2 documents for each unit of time, then it is displayed that from 2001 to 2008 there is an average of 6.75 documents for each year. During the period 2009–2012, 14, 10, 29 and 19 documents were registered respectively; during this four years, research such as the one entitled “Work in progress - Soft skills within a project - Approach based on a multimedia engineering degree” standout developed by Garcia-Panella and Badia-Corrons [31], where they introduce new methodologies supported by real projects, working with students from the Multimedia Engineering branch who simulate industrial roles in order to solve problems for clients under specific environmental conditions, but above all focused on soft skills.

Another document for these four years is the so-called “Soft Skills Recommendation Systems for IT Jobs: A Bayesian Network Approach” made by Bakar and Ting [32], where a procedure was proposed for employers to create advertisements for jobs where appropriate soft skills are identified, using the collection of data extracted from advertisement information and interviews with recognized experts. Also highlighted is the research called “Don't Panic: Improving the Social Skills of Civil Protection Workers” developed by Di Loreto et al. [33], where a serious game that was created for the improvement of social skills during crisis management is analyzed, adding fun elements during a stressful situation, and closely related to panic management; the results showed that the game is ingenious when linked to real and appropriate routines.

There is an annual growth rate of 22.99% during 2013–2017, registering this five-year period 225 manuscripts, that is, 28, 38, 43, 56 and 60 documents respectively, including the one entitled “Understanding the soft skills requirements for mobile application developers” made by Jia et al. [34], where it examines which of the soft skills are elementary in the processes of writing software on small devices; after comparing the results they concluded that developers of small devices should consider 4 soft skills not identified before, which are responsibility, fulfillment of challenging work, positive thinking with work and with the habit of coding.

Other research is highlighted for the year 2017, and is titled “Delivering Better Business Results with Social Skills” developed by Howes et al. [17], whose authors state how evolved the integration of soft skills in the training of professionals is, becoming increasingly difficult for employers and professionals; conclude that comparing case studies with surveys and evaluations with other professional services societies will guide how best to deliver business outcomes linked to social skills. Of the 79 documents registered in 2018, there is the document called “A classifier to identify soft skills in the textual description of a researcher” made by Azzini et al. [11], the objective of this study was to create a list of researchers organized according to their level of soft skills proficiency, using machine learning techniques in order to obtain a classification of the researchers' questionnaires pigeonholed in soft skills by default. Initial results around communication were promising and warrant better research in this direction.

2019, 2020 and 2021 managed to index in Scopus 97, 101 and 121 publications respectively. Of these documents is the so-called “Soft Skills Training: Performance Psychology Applied to Software Development” made by Cardenas-Castro et al. [35], that it aimed to demonstrate a training program supported by sport and performance with a psychological approach, planned for the perfection of the soft skills of a group of employees in a Colombian company that develops software; psychological skills were used as a training method but adjusted to the demands of employees and the company, showing qualitative results such as the improvement of their general well-being and social skills.

On the other hand, is the research titled “Soft skills in the training of specialists in the field of standardization, metrology and quality management as part of education for sustainable development” developed by Galimullina et al. [36], where they affirm that competencies that go beyond the professional, establish that a specialized professional competes better in the world of work fulfilling the efficient work and development of society; the educational standards of the Russian federal state were analyzed and the curricula in 20 universities that train students in the specialties mentioned in the title of this research were investigated, concluding that students claim competencies beyond the professional, and that soft skills trainings are included in curricula unevenly, while increasing student training courses in non-productive activities during the reform of the education system.

#### 3.1.3 Publications by country

**Fig. 4:**
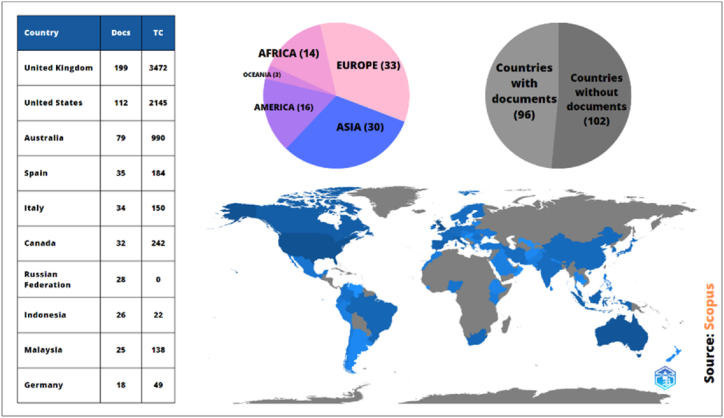
Documents by country (source: Scopus).

Fig. 4 shows the ten countries with the highest production of documents related to the variable under study. The total citations (TC) of these countries are also indicated. Likewise, the production of documents by continent and the percentage of countries without scientific production are indicated. First is the United Kingdom (n = 199), of these documents is the investigation called “Basing the design of serious games on scientific findings: the case of ENACT on training and evaluation of soft skills” produced by Marocco et al. [37], where ENACT is implemented, a serious virtual game to psychometrically evaluate the training of negotiation skills of a group of users through interaction with simulated virtual characters. Other indexed publications were found for this country [38,39].

The United States occupies the second position (n = 112), among which is the research called “Cross-cultural project-based learning and soft skills practice” made by Badets et al. [12], that it aimed to include in the curricula of students of computer science, self-reflection, and soft skills; resulting in the need for novel approaches to replicating cooperative workshops at higher levels and the provision of tools to academics and students to monitor the progress of soft skills. Several other indexed publications for this country were identified [17,40,41].

Australia ranks third in document registration (n = 79). Spain in fourth place with the highest production of documents related to the variable under study (n = 35), among them is the so-called “Teaching soft skills in Software Engineering” developed by González-Morales et al. [42], based on technical reviews of models generated throughout it, the success of this program is the motivation of the students, confirming that the students achieve a better performance and are ready for their entry into the field of software engineering. Several other indexed publications for this country were identified [8,43]. Italy ranks fifth (n = 34), Canada ranks sixth (n = 32), the Russian Federation ranks seventh (n = 28), and Indonesia ranks eighth (n = 26).

Malaysia ranks ninth in the Figure with document production (n = 25), of these investigations is the titled “Soft skills in pedagogical practices with different curriculum for engineering education” made by Mohamad et al. [44], whose objective was to identify at what level soft skills were located in higher education students, involving as a sample 302 students from different faculties of the University Tun Hussein Onn Malaysia (UTHM), the results showed that the level of soft skills is high but with a clear difference in mastery by students from different faculties. Other indexed publications were found for this country [45,46].

It is followed by Germany in tenth place (n = 18) among which is the document entitled “Advisor: analysis of a soft skills program established for students in the field of electrical engineering” produced by Ebentheuer et al. [47], whose main idea is to train and teach the new generation of electrical engineering professionals in Munich in the field of soft skills and personal technical skills, through a project with money and limited time in a safe environment, in addition to having freedom of development and performance. Several other indexed publications for this country were identified [48,49].

Of the 198 countries that make up the world, only 96 nations have managed to index studies on soft skills, that is, 48.49%. Europe leads in the production of research with the participation of 33 countries out of a total of 46, followed by Asia with the participation of 30 nations out of a total of 48. America, made up of 35 countries, follows with the participation of 16, Africa participates with 14 of its 54 nations and Oceania with only 3 nations out of 15. In terms of total citations (TC), the UK dominates with 3,472, followed by the US with 2145 and Australia with 990.

#### 3.1.4 Publications by thematic area

It should be noted that the relationship of several disciplines in the different thematic areas is accepted, consequently, the same document can be registered by more than two thematic areas immersed in the subject.

**Fig. 5:**
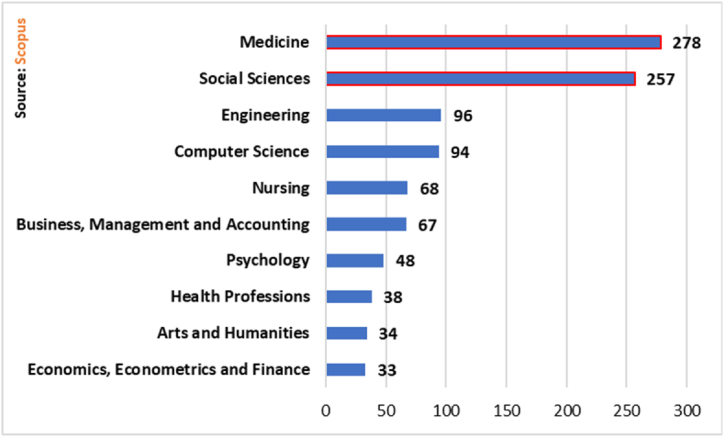
Production by subject area (scopus).

For this study, 28 subject areas were identified. In Fig. 5 only the ten areas with the most documents are indicated. In the first two places are Medicine and Social Sciences with 278 and 257 documents for each one. The studies deal with the training of non-technical skills in various acute medicine teams for technical performance and results in the operating room [50].

Engineering is in third place with 96 documents and Computer Science in fourth position with 94 publications, among which is the work entitled “A preliminary review of the scope of immersive virtual learning and training of soft skills of employees” made by Dahl [20], which gives us a review of the research literature on virtual employee training and their learning under the magnifying glass of soft skills, the result of this review shows that there is a shortage of effective studies. Other studies on this thematic area were identified [[51], [52], [53], [54]].

Nursing ranks fifth with 68 documents, followed by Business, Administration and Accounting in sixth place with 67 studies , among which is the so-called “Develop soft skills and learning outcomes of business management students in project management” produced by Md Shariff et al. [55], where the importance of adopting a project management perspective in a group of higher education students with the aim of developing communication, social skills, teamwork and leadership is discussed. Other documents in the subject area of Business, Management and Accounting were identified [12,26,35].

Psychology ranks seventh with 48 manuscripts, Health Professions in eighth with 38, Arts and Humanities in ninth with 34 and Economics, Econometrics and Finance in tenth place in Fig. 5 with 33 investigations.

#### 3.1.5 Production by Type of Publication

**Fig. 6:**
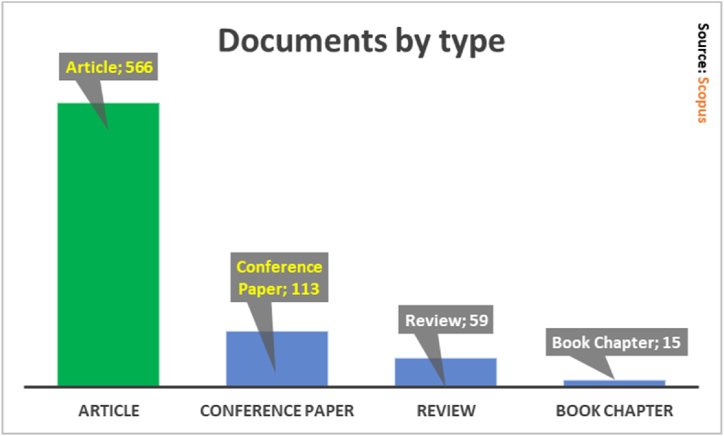
Production by type of publication.

Fig. 6 shows the 753 manuscripts of this study distributed by type of document. The “Article” leads the first place with 566 documents, in this group of manuscripts is the investigation called “Design of leadership and soft skills in educational games: the model of design of educational games of e-leadership and soft skills (ELESS)" made by De Freitas and Routledge [56], which deals with the review of the literature in relation to the types of shared leadership, presenting the ELESS model validated by the factors involved in a case study such as the leadership game, as a result we have that the ELESS model can be used to check the effectiveness of games and also to report design reports in new soft skills and leadership games. Other “Article” type documents were identified [3,5,13,26,57].

The type of document called Conference Paper ranks second with 113 studies, among them is the titled “Ideas for Adding Soft Skills Education to Service Learning and Culminating Courses for Computer Science Students” developed by Carter [58], where the social skills desired by employers are studied and the possible inclusion of those skills in a learning course for a group of students from Point Loma Nazarene University, in the end suggest the possible modification of future courses in soft skills. Another featured Conference Paper is the so-called “Soft Skills in Software Development Teams: A Survey on the Views of Leaders and Team Members” produced by Matturro et al. [16], where 35 software engineering experts from an Uruguayan company are interviewed to find out which soft skills, they consider most valuable for the leader and the members of the group. They conclude that “communication”, “leadership”, “customer service” and “teamwork” are the most valuable for the team leader, vastly different from the team members, who value “motivation”, “commitment”, “teamwork” and “problem solving”. Other documents of the type “Conference Papers” were found [12,25,28,40,42,45,54].

The type of document called “Review” registers 59 publications in third place [22]. The “Book Chapter” ranks fourth with 15 documents [[59], [60], [61], [62]].

### 3.2 Most explored topics

**Fig. 7:**
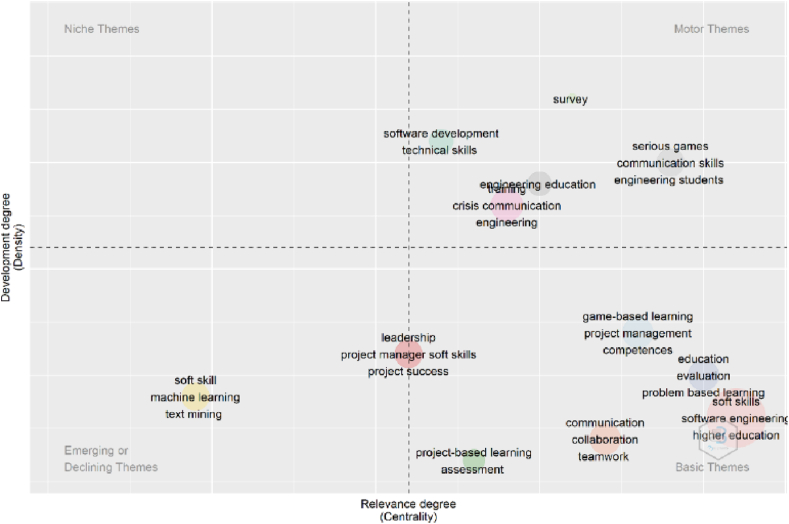
Thematic map.

The Thematic Map (Fig. 7) generated by the RStudio software is divided into 4 quadrants and each represents a category: basic themes, motor themes, niche themes and emerging or declining themes. The themes reflected in this map are identified in clusters that orbit according to the Degree of Relevance (Centrality) and the Degree of Development (Density). The default parameters for the generation of the Thematic Map were the author keywords (n = 250) and minimum cluster frequency per thousand documents (n = 5). According to the above, it was possible to identify 11 subjects with greater linkage to the variables under study and these are shown in Table 1.

**Table 1:** Themes identified in the Thematic Map.

No THEMATIC	DESCRIPTION OF THE THEME	THEMATIC CATEGORY	DEFINITION OF THE CATEGORY
1	“Soft skills in software engineering at the higher education level”	Basic Theme	Themes caused by critical situations that affect our society
2	“Soft skills and communication”	Basic Theme	
3	“Soft skills and engineering education”	Motor Theme	Main themes of the research front
4	“Soft skills in virtual environments”	Motor Theme	
5	“Soft skills in machine learning”	Emerging Theme	New topics that are being studied more and more
6	“Serious games in teaching soft skills”	Motor Theme	
7	“Soft skills for problem-based learning”	Basic Theme	
8	“Soft skills for project management”	Basic Theme	
9	“Soft skills and technical skills”	Motor Theme	
10	“Project-based learning for the assessment of soft skills”	Basic Theme	
11	“Soft leadership skills”	Basic Theme	

#### 3.2.1 Soft skills in software engineering at the higher education level

It is considered as a basic theme as indicated in Table 1 and covers publications related to the introduction of soft skills in students or teachers who develop software in higher education. In this topic is the research entitled “Identification based on soft skills processes of the software project manager” carried out by Pinkowska et al. [63], based on the study of the processes linked to the qualities of people to manage software projects and the consequent description of the soft skills, attributed to the project manager for the execution of said processes. Other studies related to this theme were found [35,57].

#### 3.2.2 Soft skills and communication

According to Table 1, it is considered a basic theme and includes publications about soft skills in communication. People with these skills work well in teams, are focused on results and are resilient in their social interactions. One of the documents related to this topic is the so-called “How to stimulate SoSE engineers to develop interpersonal skills? How effective is a conference in Nonverbal Communication?” This conference paper was developed by Charite and Muller [15] and argue that the human content of the system of systems (SoS) is relatively significant. The participation of individuals and groups within an SoS manages to influence the engineering process. They emphasize that engineers require soft skills to work effectively in the Area of SoS. These authors studied the effect that a lecture could have on so-called nonverbal communication as a resource to encourage systems engineering students in Kongsberg, Norway, to progress even better in their soft skills. The findings found that students have little knowledge about the expressions that make up nonverbal communication. Other documents related to this theme were found [58,64,65].

#### 3.2.3 Soft skills and engineering education

It is a motor theme according to Table 1 and deals with publications where they highlight the importance of applying soft skills in the education of engineers to acquire a balanced training within technical and non-technical resources. A study on this topic is called “Preparing high school students for college while training engineering students in interpersonal skills” conducted by Schwartz [41], who describes how and why he has used the high-impact practice in service training to support high school students in decreasing skills gaps. It also describes the strategy used by students in the introductory programming course to solve verbal problems that require more reflection, who then pose them as group work to high school students at the end of the semester. Finally, high school and college students discuss the jobs providing an interesting and transcendental experience. Another document related to this theme was identified [66].

#### 3.2.4 Soft skills in virtual environments

It is a motor theme according to Table 1 and shows publications on the soft skills adopted in virtual environments such as time management, critical thinking, problem solving, communication, teamwork, leadership, adaptability, proactivity, motivation, responsibility, among others. One research that deals with this topic is the so-called “Use of simulated digital role plays to teach ‘soft skills' of healthcare” developed by Schutt et al. [67], whose objective was to synthesize the work on virtual soft skills executed and analyzes the drawbacks identified by the development groups that work in the medical area. The first section provides historical reviews of the phrase “interpersonal skills” and the related digital training activities. It ends with a case study of a multidisciplinary team of Australian educators, researchers and software developers who have managed to design a range of virtual healthcare products.

#### 3.2.5 Soft skills in machine learning

According to Table 1, it is an emerging theme and covers publications related to the important soft skills in the data scientist to be able to solve problems effectively in machine learning. Machine learning requires professionals capable of teamwork and flexible, as well as having analytical and critical thinking, creativity, empathy, assertive communication, and emotional intelligence. A document related to this topic is the one called “The application of mathematical thinking methods in the optimization of projects for the development of interpersonal skills” developed by Li et al. [68], which begins by defining the term “central competitive power” and then presents how mathematical thinking forms would be applied in the optimization of professional specialization projects. Also, numerous thematic training events are developed for the linking of students at a higher level and thus be able to increase the result of these activities. Other documents related to this theme were found [3,11].

#### 3.2.6 Serious games in teaching soft skills

According to Table 1, it is a motor theme and covers publications related to the application of serious games or formative games in the teaching of soft skills in areas such as education, defense, politics, religion, engineering, among others. A study on this topic is the so-called “Use of online multiplayer games for the teaching of soft skills in higher education” prepared by Pagel et al. [49], where an elective course developed to instruct soft skills in engineering subjects using the multiplayer game called EVE Online is analyzed. The assessment tools consist of an online questionnaire on social presence, expectation, and internal motivation, as well as semi-structured interviews. The assessment allows multiplayer-virtual games to become potential soft skills training mechanisms aimed at students specializing in digital games. Other documents related to this theme were found [67,69,70].

#### 3.2.7 Soft skills for problem-based learning

It is raised as a basic theme according to Table 1 and covers publications that address soft skills and problem-based learning for the creation of procedures that solve curiosities, questions, and doubts about complex problems of life. A document related to this topic is the so-called “PTBL: a learning model based on PBL and TBL for the training of soft skills with the support of the 3D virtual pedagogical platform (3DVPP)" carried out by Chen et al. [71], who propose a new learning model that incorporates task-based learning, problem-based learning, and Web3D technologies; such a model could improve teamwork skills in the area of software engineering and leave behind the frequent difficulties of the normal course. Likewise, it proposes two courses with this new model: virtual meeting course in 3D and course of game of tasks. In addition to these courses, several results of the experiments carried out in the two case studies in students and professors of the Department of Software Engineering of Tsinghua University are presented. Other documents related to this theme were found [47,72].

#### 3.2.8 Soft skills for project management

Identified in Table 1 as a basic theme and covers publications around soft skills in project management. They are a set of skills suitable for the management, coordination, execution, and supervision of a viable project. A study on this subject is the so-called “Soft skills and the construction manager: The professional chameleon” developed by Van Heerden [73], whose objective was to identify a profile for the typical construction manager in South Africa according to the current requirements of the industry. Case studies were included to show that Melvin's profession guide was the product of 11 common soft skills and could be represented by Bono's 6 thinking hats. A bibliographic analysis was carried out for each soft skill assumed. According to this study, a construction student has some soft skills that could be developed when working with Bono's 6 thinking hats as a manager, increasing his effectiveness on site. Other studies related to this theme were identified [14,74].

#### 3.2.9 Soft skills and technical skills

It is shown as a motor theme within Table 1 and includes publications where soft skills are linked to technical skills that are knowledge and skills that guarantee the execution of specific activities. They are usually combined with computer, mechanical, scientific, or mathematical tasks. Many cases include software development, knowledge of programming languages, mechanical equipment, among others. An article on this topic is called “Which are more difficult? Soft skills or hard skills?” prepared by Pieterse and Van Eekelen [75], who detail a number of technical skills and professions fundamental to students' success in their software development career; the authors conduct studies aimed at understanding students' concerns about strengthening these skills. They show their techniques to highlight skills gaps and thus intervene and propose review processes. The creation of alternatives for the improvement of students' skills is discussed according to their experiences and the conclusions resulting from this study. Other studies related to this theme were identified [4,26,51].

#### 3.2.10 Project-based learning for the assessment of soft skills

It is a basic theme according to Table 1 and covers publications related to soft skills assessments through project-based learning, which is a dynamic educational procedure where the student is the leader and learns by resorting to projects that are developed outside the classroom. An investigation on this topic is the so-called “Design and Development of an Educational Game for Leadership Assessment and Optimization of Soft Skills” carried out by Chatziantoniou et al. [76], whose objective was to design a game that simulates real scenarios for the formation of skills suitable for leadership and the professional area. The driver used for the design of this game was the Unity 3D engine, in addition to the narration tool and the novel Fungus. With this process, a two-dimensional game was put into practice that empowers the player to participate and in turn strengthen skills focused on leadership that in the future will help him in the professional area. Other studies related to this theme were identified [12,62].

#### 3.2.11 Soft leadership skills

Considered a basic theme within Table 1 and represents publications that deal with the learning of soft skills in leaders who know how to plan and manage human capital, in addition to this they must have a general conception and collaborative attitude. A Conference Paper related to this topic is the so-called “Interpersonal relationships, leadership and other interpersonal skills in software development projects: a systematic review” developed by Elizalde and Bayona [77], where the objective of this review of 23 important documents was to know how interpersonal relationships, non-technical skills and constructs are linked with the management of Software Development projects. These articles were evaluated systematically, requiring considerable links between the positive management of software development projects and psychological variables. Other studies related to this theme were identified [27,76].

### 3.3 Future research agenda

**Fig. 8:**
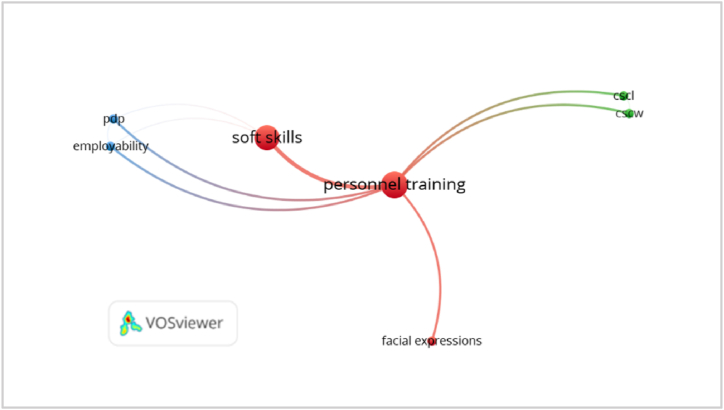
Keywords with the least co-occurrence.

To develop an agenda with potential topics for future research, the 1057 keywords contained in the 753 documents were discussed, leading to the derivation of five (5) areas that could be explored by the authors in their future research. The criteria used for the selection of these topics is the keywords with the least co-occurrence (Fig. 8), i.e., collaborative work (n = 2), computer-aided collaborative work (n = 2), facial expressions (n = 2), employability (n = 2) and Professional Development Plan (n = 2).

Table 2 below shows the five possible research topics. Each of these is discussed below the table.

**Table 2:** Potential research topics.1)Soft skills in collaborative work (CSCL): These are studies on soft skills in collaborative work, i.e., when two or more individuals collaborate by exchanging opinions, attitudes, and skills for the achievement of a common goal.2)Soft skills in computer-aided collaborative work (CSCW): Studies in this topic deal with soft skills in computerized co-working, where employees can cooperate in work-related activities through common computer networks, including software and processes that provide enhancements, changes, and data during runtime.3)Facial expressions as a mirror of soft skills: It deals with studies related to facial language as a reflection of soft skills, this being one of the most common forms of non-verbal communication.4)Soft skills for employability: These are studies that deal with soft skills for employability, i.e., a total of essential skills and attitudes in different jobs. Likewise, it is called “soft skills”, “fundamental skills”, or “training skills” for employment.5)Professional Development Plan (PDP) to assess soft skills: Studies that deal with professional growth plans, which are documents that evaluate existing skills and these help to determine the objectives of a profession, promote plans and identify resources to achieve goals.

#	Future research topics	Author with document linked to the topic
1	Soft skills in collaborative work (CSCL).	[78,79]
2	Soft skills in computer-aided collaborative work (CSCW).	[80]
3	Facial expressions as a mirror of soft skills	[81]
4	Soft skills for employability	[82]
5	Professional Development Plan (PDP) to assess soft skills	[17]

## 4 Limitations

Despite the importance of the contributions of this bibliometric research, the following limitations should not be overlooked. The first, the use of a single database such as Scopus to obtain the information for this study. It is well known that using multiple databases provides better reach, but this single database contains numerous documents, many of which are indexed in other databases in parallel.

The second, the number of terms used to search for documents related to the topic in question. Despite entering several specific terms related to the topic of study, other synonyms could have been left out, consequently, other documents could have been left out of the set of studies collected.

## 5 Conclusions

To answer the first question of this research, it was detected that the keyword with the highest co-occurrence is personnel training (n = 110) with a close relationship with soft skills (n = 79). It was also evidenced that 2021 (n = 121) was the year with the highest production of documents. The United Kingdom, with 199 documents, is the country with the most publications, but it was found that 51.52% of all countries have not investigated the subject of this study. Medicine and Social Sciences are the thematic areas that most investigate the subject of this study, registering 278 and 257 manuscripts each. The Article is the type of document with the most studies (n = 566), followed by Conference Papers (n = 113), the sum of these two represents 88.84% of the 753 selected studies, that is, documents with original information, which means valuable information for this bibliometric study.

To answer the second question, it was possible to identify 11 topics of greater exploration with the variables under study (Table 1), of which 6 are categorized as “basic theme”, 4 as “motor theme” and 1 as “emerging theme".

To answer the third question of this research, after discussing the information generated by the two-software indicated in the methodology section, it was possible to derive 5 potential topics for future research. Among them: “Soft skills in collaborative work (CSCL)”, “Soft skills in computer-aided collaborative work (CSCW)”, “Facial expressions as a mirror of soft skills”, “Soft skills for employability” and “Professional Development Plan (PDP) to assess soft skills” (Table 2).

Although soft skills are the essential component for organizations when training staff, we found a large gap in the literature on this topic in terms of the production of documents by country during the period 1999–2021. This bibliometric study is one of the most up to date, becoming important research because it provides information that can be used as a guide in future research.

It is suggested to carry out research with the topics little explored, such as the one indicated in Table 1 as an “emerging theme” and the five indicated in Table 2 as an agenda for future research. The data included in this bibliometric review is updated until December 2021 and only the Scopus database was used.

## Author contribution statement

All authors listed have significantly contributed to the development and the writing of this article.

## Data availability statement

Data included in article/supp. material/referenced in article.

## Additional information

No additional information is available for this paper.
